# Thermodynamically Stable Functionalization of Microporous Aromatic Frameworks with Sulfonic Acid Groups by Inserting Methylene Spacers

**DOI:** 10.3390/molecules29071666

**Published:** 2024-04-08

**Authors:** Simon F. Winterstein, Michael Bettermann, Jana Timm, Roland Marschall, Jürgen Senker

**Affiliations:** 1 Inorganic Chemistry III and Northern Bavarian NMR Centre, University of Bayreuth, Universitaetsstrasse 30, 95447 Bayreuth, Germany; 2 Physical Chemistry III, Department of Chemistry, University of Bayreuth, Universitaetsstr. 30, 95447 Bayreuth, Germanyroland.marschall@uni-bayreuth.de (R.M.)

**Keywords:** covalent organic framework, porous aromatic framework, synthesis, side chain, proton conduction

## Abstract

Porous aromatic frameworks (PAFs) are an auspicious class of materials that allow for the introduction of sulfonic acid groups at the aromatic core units by post-synthetic modification. This makes PAFs promising for proton-exchange materials. However, the limited thermal stability of sulfonic acid groups attached to aromatic cores prevents high-temperature applications. Here, we present a framework based on PAF-303 where the acid groups were added as methylene sulfonic acid side chains in a two-step post-synthetic route (SMPAF-303) via the intermediate chloromethylene PAF (ClMPAF-303). Elemental analysis, NMR spectroscopy, electrochemical impedance spectroscopy and X-ray photoelectron spectroscopy were used to characterize both frameworks and corroborate the successful attachment of the side chains. The resulting framework SMPAF-303 features high thermal stability and an ion-exchange capacity of about 1.7 mequiv g^−1^. The proton conductivity depends strongly on the adsorbed water level. It reaches from about 10^−7^ S cm^−1^ for 33% RH to about 10^−1^ S cm^−1^ for 100% RH. We attribute the strong change to a locally alternating polarity of the inner surfaces. The latter introduces bottleneck effects for the water molecule and oxonium ion diffusion at lower relative humidities, due to electrolyte clustering. When the pores are completely filled with water, these bottlenecks vanish, leading to an unhindered electrolyte diffusion through the framework, explaining the conductivity rise.

## 1. Introduction

The transport of protons in and through a solid material is a topic that occurs in many applications such as sensors, ion-exchange desalination, CO_2_ converters, electrolyzers, and fuel cells [1,2]. The latter devices are receiving more and more attention, due to their broad possible uses [3,4]. In particular, the polymer electrolyte membrane fuel cell (PEM-FC) is discussed for application in mobile devices [4,5,6]. Although PEMFCs are already commercially available, they still need to be further developed [6]. Many types of research focus on the membrane electrode assembly (MEA) [7,8,9], which constitutes the main part of a PEM-FC [10]. It contains the diffusion layers for gas transport, electrodes with the catalyst for the chemical reaction, and the membrane for the proton transport between the two electrodes [11]. In addition to proton transport, there are other requirements placed on the membrane, such as electrical isolation, high thermal stability, and chemical inertness [12].

Despite the research into the MEA, Nafion^®^ and its derivatives are the material of choice [7]. Nafion^®^ is an inert perfluorinated copolymer with a high proton conductivity of up to 0.1 S cm^−1^ at 353 K and 100% RH [13]. Despite its excellent proton conductivity, Nafion^®^ contains disadvantages like high production costs, harmful degradation products, and costly disposal or waste management [14,15,16]. Moreover, the working temperature of Nafion^®^ is limited to 253 K and 353 K [17,18]. The higher limit is caused by the low retention force that water exhibits in the mesopores of Nafion^®^ [19]. Thus, it is possible to remove the bulk-like water in the inner pore space at higher temperatures quite easily. At the lower temperature limit, water crystallizes, resulting in a strong decrease in the proton conductivity [19,20]. This leads to a complicated water management system for running a PEM-FC with Nafion^®^ to keep a constant hydration level at the operating temperature [21,22].

To increase the confinement and stability of the proton-conducting membrane, several strategies are pursued. Among other things, composite materials consisting of Nafion^®^ are being developed [23,24,25]. The introduced materials such as metal oxides, acids, metal–organic frameworks (MOFs), and covalent organic frameworks (COFs) increase the uptake of water and the adsorption enthalpy, to increase the operating temperature range. Another focus of research is to find new materials for proton conduction. Porous materials such as carbon materials [8], silica [9], organosilicates [9], MOFs [26,27], and COFs [28,29] with acid groups were developed and investigated according to the ion-exchange capacity and the proton conduction. The introduced sulfonic acid groups led to strong interactions with the adsorbed water molecules [20], high ion-exchange capacities (IECs) and excellent proton conductivities. We already reported sulfonated porous aromatic frameworks (SPAFs), which have an amorphous structure and contain a high ratio of accessible micropores [30,31], whereas the interpenetration of the structure rises with increasing numbers of phenyl rings in the spacers [32,33]. Introducing SO_3_H groups to SPAF-2_0.8_, enabled a six-times higher IEC than Nafion^®^ [31]. Please note that the PAF framework of the SPAF-2 equals the one used for this work but was renamed PAF-303 for consistency with the recent literature [34]. The high charge concentration combined with the adsorbed water phase enables a proton conductivity of about 1 S cm^−1^. This is supported by water, with a high diffusion coefficient of the water molecules being stored in micropores [31]. Moreover, the sulfonic acid groups are bonded directly to the framework backbone, which leads to a reversible bond, known in organic chemistry as a protection group [35]. This limits the SPAF frameworks in their use in high-temperature applications.

To enhance the thermal and chemical stability of a sulfonic acid proton conductor, the Das group introduced aliphatic C3 and C4 side chains with a terminal sulfonic acid group to UiO-66 [36]. They observed for the C3 side chain the higher conductivity of 1.64 × 10^−1^ S cm^−1^, caused by the lower pK_A_ value, due to the shorter chain length. Moreover, these MOFs, especially the CH_2_-SO_3_H bond, showed excellent thermal and chemical stability. Our aim in this work is to follow this strategy and synthesize a porous aromatic framework that contains sulfonic acid groups that are bound to the framework via an aliphatic side chain. For this, we developed a synthetic strategy that post-synthetically introduces methylenesulfonic acid side chains into PAF-303. The synthesized frameworks were characterized by infrared (IR) and nuclear magnetic resonance (NMR) spectroscopy, elemental analysis (EA), energy-dispersive X-ray spectroscopy (EDX), X-ray photoelectron spectroscopy (XPS), Ar and water physisorption, thermal gravimetric analysis (TGA), differential scanning calorimetry (DSC), and electrochemical impedance spectroscopy (EIS). 

## 2. Results and Discussion

**Scheme 1:**
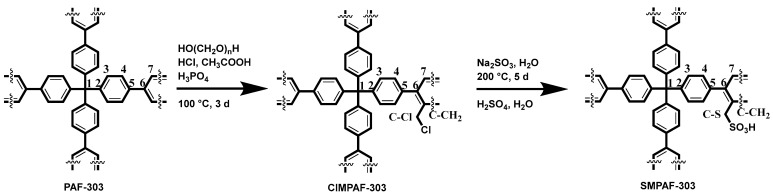
Synthesis of sulfonic acid methylene PAF-303 (SMPAF-303) originating from PAF-303 with the intermediate chloromethylene PAF-303 (ClMPAF-303).

The synthesis of the methylenesulfonic acid porous aromatic framework (SMPAF-303) was a three-step synthesis starting with the formation of the porous aromatic framework (PAF-303). Second, the methylenechloride group was introduced by a Blanc reaction to the methylenechloride porous aromatic framework (ClMPAF-303) (Scheme 1). Finally, the functional group interconversion (FGI) of the methylenechloride group into the methylenesulfonic acid group leading to the porous aromatic framework (SMPAF-303) was performed.

**Figure 1:**
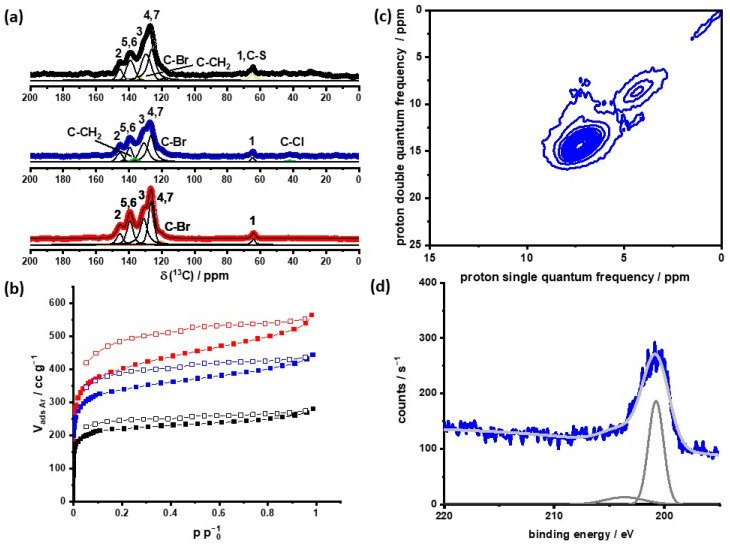
(**a**) SP ^13^C MAS NMR spectra of PAF-303 (red), ClMPAF-303 (blue), and SMPAF-303 (black) with corresponding deconvolution and assigned carbon atoms of each structure. (**b**) Ar physisorption measurements of PAF-303 (red), ClMPAF-303 (blue), and SMPAF-303 (black). The full squares represent the adsorption and the empty ones the desorption. (**c**) Double quantum ^1^H solid-state NMR of ClMPAF-303. (**d**) Cutout of the XPS spectra of ClMPAF-303 (Figure S3, ESI) showing the Cl 2p signal with the fitting of the two overlapping signals (gray).

The PAF-303 was synthesized according to the protocol of Kaskel et al. [37]. The framework is formed by the 3D-linker tetrakis-(4-bromophenyl)methane and the linear spacer 1,4-phenylenedibronic acid. These two molecules are coupled by a Suzuki cross-coupling reaction with a palladium catalyst. The successful formation of PAF-303 is shown in the IR spectrum (Figure S1, ESI) by the aromatic stretching vibration at 515 cm^−1^ and the para-substituted phenyl out-of-plane bending vibration at 807 cm^−1^, combined with the absence of the para-substituted ring and stretching vibration of C-Br at 1077 cm^−1^ and the broad stretching vibration of O-H of the boronic acid group at about 3300 cm^−1^. Moreover, the IR spectrum of PAF-303 does not contain the two overlapping signals of the B-OH vibration and B-Ar out-of-plane deformation vibration, at about 700–800 cm^−1^. Additionally, the resonances 5 and 6 at 139 ppm in the single-pulse ^13^C NMR spectrum (Figure 1a) relate to the sp^2^-hybridized carbon demonstrating that the C-C coupling was successful. For the quantification of the cross-coupling degree, the ratio of C-Br (120 ppm) to the C-1 (64 ppm) signal was calculated by deconvolution of the single-pulse (SP) ^13^C MAS NMR spectra, leading to a cross-coupling degree of 85%, calculated by the 0.6 end groups, for four possible bond formations (Table S1, ESI). Combined with the EA and the EDX measurements (Table S2, ESI), the Suzuki cross-coupling led to a crosslinking degree of 86% and a stoichiometry for PAF-303 listed in Table 1.

The irreversible bond formation of the Suzuki cross-coupling reaction led to a partial interpenetration for the amorphous framework (Figure S2, ESI), which is already mentioned for PAF-1 by simulation [32,33] and experimentally observed for PAF-303 [31,37]. This interpenetration forms a high number of micropores, which are shown in the strong adsorption of Ar at small partial pressures (<0.05) (Figure 1b). At higher partial pressures the volume increases linearly, with a strong hysteresis over the whole pressure range for desorption. This behavior is typical of a flexible microporous framework like PAF-303. The calculated pore-size distribution from the physisorption measurement gives a broad microporous distribution of pore sizes and a total number of micropores of about 74% (Table 2).

This high interconnecting pore space and surface area, where the linker units are well accessible, allows for further reactions [38,39,40,41] such as introducing acidic units for proton conduction [30,31]. To achieve the introduction of these chemical- and temperature-stable proton-conducting units in the framework, PAF-303 reacts with formaldehyde and HCl to the intermediate ClMPAF-303 (Scheme 1). The formation of the C-CH_2_ bond is corroborated by the two additional signals in the solid-state SP ^13^C NMR spectrum (Figure 1a) at 41 ppm for the aliphatic side-chain carbon (CH_2_-Cl) and 136 ppm for the aromatic carbon to which the side chain is connected. The deconvolution of the SP ^13^C MAS NMR spectrum (Figure 1a) gives a ratio for the C-1- to CH_2_-Cl signal of about one-to-one and for C-1 to the aromatic carbon of about 1.8 (Table S1, ESI). Combined with the results of the EDX measurements, an average of one side chain per repeating unit of the framework (Table 1) was derived. Moreover, the elemental analysis agrees with the observation from NMR spectroscopy and EDX measurement (Tables S1 and S3, ESI). 

The measured DQ-SQ MAS ^1^H NMR spectrum (Figure 1c) shows self-correlation signals of the aromatic and aliphatic protons. Additionally, cross-correlation between aromatic and aliphatic protons were observed, confirming that the methylene chloride groups are attached to the aromatic linkers. Furthermore, the X-ray photoelectron spectrum exhibits the carbon and bromine signals that belong to the backbone as end groups (mentioned before), which indicate the stability of the backbone of the framework during the synthesis (Figure S4, ESI). Additionally, a chlorine signal is observed (Figure 1d). The Cl 2p peak splits into two peaks at about 200 and 203 eV. These energies are typically for organic chlorines. Both methods show the successful addition of the formaldehyde to the aromatic unit, and the following conversion to the functional group chloromethylene. In contrast, the IR spectrum (Figure S1, ESI) does not change significantly compared to those of PAF-303, which suggests a small amount of chlorine compared to the number of carbon atoms in the repeating unit.

To introduce sulfonate groups into the framework, the chlorine groups were replaced by SO_3_Na. Subsequently, a sodium–proton ion exchange re-established the acid groups. The SP ^13^C NMR spectrum (Figure 1a, Table S1) shows that the chloromethylene side chains could be transformed quantitatively. Further, it shows an overlapping signal of C-1 at about 65 ppm, which belongs to the quaternary carbon of the 3D linker and the carbon of the CH_2_-SO_3_H side chain. The quaternary aromatic carbon, where the side chain is connected to the backbone, is at about 130 ppm. Additionally, EDX spectroscopy, EA, and titration (Tables S4 and S5, Figure S4a, ESI) were carried out. On average, one sulfonic acid methylene side group per repeating unit could be introduced, leading to an IEC of 1.73 meq g^−1^ (Table S5, ESI). This IEC is twice as high as the one for Nafion^®^ (0.9 meq g^−1^) [42]. The sulfonation degree x is defined as the number of sulfonic acid groups per benzene rings of the repeating unit. This confirms the stoichiometry of SMPAF-303 with about one sulfonic acid group per repeating unit.

From PAF-303 over ClMPAF-303 to SMPAF-303 the thermal-oxidative stability decreased (Figure S5, ESI). PAF-303’s decomposition sets in at about 590 K, which represents the breaking of C-C bonds in the backbone. After chloromethylation, the curve changes in a stepwise decomposition where the first weight loss starts at 480 K, followed by a bigger loss at about 590 K. We assume that the first weight loss is attributed to the side chains, in line with the calculated weight ratio of the side chain to the backbone (one to nine), and that the second one is caused by the decomposition of the framework. SMPAF-303 exhibits a continuous weight loss up to 400 K, which is typical for water loss from a hydrophilic framework (Table 1). The side-chain decomposition sets in at about 500 K. followed by the backbone, as for ClMPAF-303. The first decomposition fits very well with the percentage weight of about 15% of the side chain compared to the whole framework weight. This high-temperature stability compared with the irreversibly bonded sulfonic side chains (Figure S4b and Table S5), which are stable under harsh conditions, offers an ideal basis for use as material for proton conduction in high-temperature fuel cells.

For high-temperature fuel cells, it is necessary to build up a strong confinement. Two parameters influence the confinement strongly. First, the interaction between the sulfonic acid groups and the water molecules, and second the pore size. For the latter, a smaller pore size increases the confinement. Due to the introduction of side chains, the amount of adsorbed gas is reduced, and the corresponding hysteresis becomes smaller (Figure 1b). This trend is also shown in the pore volume, which decreases to about 740 m^2^ g^−1^ (DFT surface area 773 m^2^ g^−1^) and gives a pore volume of 0.337 cc g^−1^ (Table 2). The introduced side chains reach into the pore, which causes the reduced pore volume (Figure S6). This is already seen in the literature in the post-synthetic modification of PAF by the introduction of other side chains [38,39,43,44].

**Figure 2:**
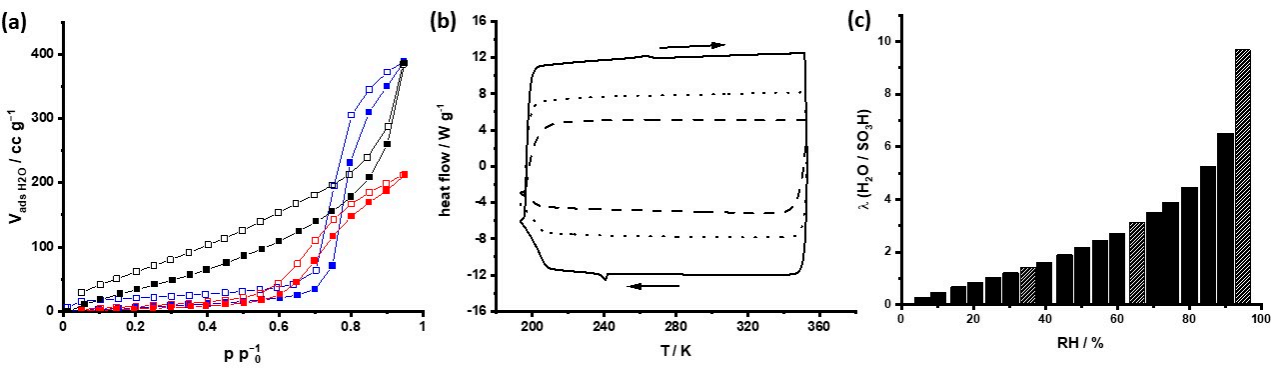
(**a**) H_2_O physisorption measurement of PAF-303 (blue), ClMPAF-303 (red), and SMPAF-303 (black) at 298 K. The full squares represent the adsorption and the empty ones the desorption. (**b**) DSC measurements of SMPAF-303 at 33 (dashed), 67 (dotted), and 100 (lined) % RH. (**c**) λ, calculated from H_2_O physisorption measurement, against relative humidity. The relative humidities of 33, 67, and 98% are highlighted by hatching.

PAF-303, as well as ClMPAF-303, shows a hydrophobic behavior with a gate opening at 70 and 60% RH (Figure 2a). These trends are caused by the strongly hydrophobic nature of the frameworks compared with the microporosity. The introduction of chloromethylene side chains leads to a decrease in the total water uptake by a factor of roughly two. After substituting chlorine with sulfonic acid groups, the isotherm exhibits a type-II behavior [45]. So, the framework changes its hydrophobic surface into a partially hydrophilic surface, which means a hydrophobic backbone with a hydrophilic side chain that strongly interacts with the adsorbed water molecules. That is shown in the water uptake of SMPAF-303, which increases steadily with higher relative humidity. SMPAF-303 finally reaches the same uptake as PAF-303. This indicates the stronger confinement effect compared to ClMPAF-303 and PAF-303. SMPAF-303 adsorbs the same amount of water with a smaller pore volume than PAF-303 and more water than ClMPAF-303. This strong confinement is shown in DSC measurements (Figure 2b) for samples equilibrated at 33 and 67% RH. At these relative humidities, no freezing and melting of the adsorbed water was observed. For 100% RH, the DSC curves of SMPAF-303 show a melting point with an onset of 256 K (Table S6, ESI). This is typical for 20% of the adsorbed water being stored in inter-particular spaces and mesopores. This is in line with the determined micro- and mesopores calculated from the Ar physisorption measurement and DFT calculations of the pore space (Table 2). Instead, Nafion^®^ contains meso- and macropore water, which leads to the freezing of about 40% of water at about 263 K [19,46]. This large amount of non-freezable water results in a limitation of the working temperature, which is already known in the literature [17,18].

SMPAF-303 is able to store about 31 wt.-% of water. A total of 80% of the water is absorbed in micropores. The limited uptake of water due to the uptake of PAF-303 is caused by the fact that the framework contains, next to the hydrophilic side chains, the hydrophobic backbone, which limits the total water uptake. The number of water molecules per sulfonic acid group (λ) is a value for the comparison of proton-conducting materials. SMPAF-303 has up to about 10 H_2_O/SO_3_^−^ for 100% RH. With decreasing RH, λ is also reduced, with an exponential trend (Figure 2c). The state-of-the-art material Nafion^®^ [46] has about 14 water molecules per sulfonic acid group. This means that the charges of the hydroxonium ions are influencing each other much more in SMPAF-303 than in Nafion^®^.

**Figure 3:**
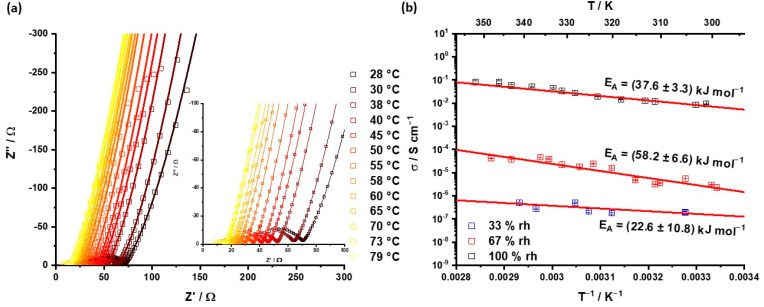
(**a**) Nyquist plot of SMPAF-303 at 100% RH with an enlarged image section of the semicircle of the bulk proton conductivity. (**b**) Arrhenius plot of the conductivity of SMPAF-303 EIS measurements at 33 (empty), 67 (half-filled), and 100 (full) % RH with a linear fit and the calculated activation energy.

The high charge-carrier concentration (1.83 meq g^−1^) at a relatively low λ, the strongly adsorbed water molecules up to 31 wt.-%, and the irreversible bonded sulfonic acid groups make SMPAF-303 a promising proton-conduction material. To determine the proton conduction at a temperature range from room temperature to 353 K, the powder was saturated under 33, 67, and 100% RH and pressed to a pellet. The EIS spectra were simulated with an equivalent circuit containing one preliminary resistance (R_E_) and three RC elements in series (Figure S7, ESI). This type of equivalent circuit is typically used for water-mediated microporous proton conductors, which are placed in a parallel-plate capacitor-like setup [31,47]. In the used equivalent circuit, the R_E_ simulates the connection of the cables to the measurement setup. This element is negligible at larger impedances, such as for the measurements for 33% RH and 67% RH. For all relative humidities, the first two semicircles belong to bulk and grain boundaries, and the third one to the electrode interface (Figure 3 and Figure S8–S10, ESI).

Saturating at 33%, RH leads to a high protonation level of water, where two-thirds of the water molecules are protonated and λ is 1.4, which leads to strong ion interactions between the oxonium ions and the SO_3_^−^ groups, leading to strong ion-pair interactions. Additionally, the movement of protons is limited in the bulk and in the grain boundaries, which results in the same order of capacities for both RC elements (Table S7, ESI). For λ equal to 3, which is relised when saturating at a relative humidity of 67% RH, the separation between the bulk- and grain-boundary semicircles becomes visible. This higher water content results in an unsaturated solvation shell of the oxonium ions and separates the charges from each other. At 100% RH there is a clear separation between the bulk- and grain-boundary conductivities, as well as of the electrode (Figure 3a). Here, λ is about 10, which enables a free water diffusion. This leads to a better solvation of oxonium ions, resulting in higher shielding of the opposite charges to each other than for 67% RH. This stronger separation increases the mobility of the charged carriers shown by lower resistances (Table S8, ESI). The low resistances induce the conduction resistance of the electrode to be visible as electrode resistance (Figure 3a).

The conductivities of the three relative humidities are calculated from the bulk resistances. SMPAF-303 reaches conductivities from 10^−7^ to 10^−1^ S cm^−1^ (Figure 3b). For 33% and 67% RH, bulk- and grain-boundary conductivities are on the same order of magnitude (Tables S7 and S8, ESI). This behavior is untypical, and can be explained by an unsaturated solvation shell of the oxonium ions in the pores and strong ion interactions, which lead to an immobilization of the charges. Additionally, we suggest alternating hydrophilic and hydrophobic areas. The hydrophilic regions are close to the side chains, and the hydrophobic regions are the aromatic backbone without side chains.

In contrast, at 100% RH, bulk- and grain-boundary conductivities increase strongly (Table S9, ESI). We attribute this to the higher λ (Figure 2c) rather than to lower humidities. About 10 H_2_O molecules per sulfonic acid group lead to a sufficient hydration shell of the oxonium ion and, concomitant with the separation of this ion, to their counterion. This results in a high conductivity of 0.1 S cm^−1^ at 350 K and lower activation energy, compared to lower humidities of about 38 kJ mol^−1^ (Figure 3b). This high conductivity, which is in the same order as Nafion^®^ [13], suggests a free diffusion of water molecules and oxonium ions through the framework, which is typical for microporous materials [31]. Compared to SPAF-2, SMPAF-303 is one decade lower [31]. This difference results in an alternating polarity in the framework structure, which hinders the free diffusion in SMPAF-303, compared to SPAF-2. We suggest that the conductivity can be increased with a higher side-chain concentration to form a constant diffusion pathway through the framework.

## 3. Material and Methods

### 3.1. Materials

All chemicals and solvents which are used for the synthesis were commercially available and were of reagent grade, without further purification. 1,1′-Bis(di-isopropylphosphino)ferrocene (DIPPF, 98%) was purchased from abcr. 1,4-Phenylenediboronic acid (BDB, >97%) and tetrakis(triphenylphosphino)palladium(0) (Pd(PPh_3_)_4_, >97%) were purchased from TCI. Glacia acid (conc.), sodium sulfite (>95%), and sulfonic acid (95–97%) were purchased from Merck KGaA. Ethanol (99.3%) was purchased from VWR-Chemicals. Potassium carbonate (>99%), paraformaldehyde (95%), and zinc (II) chloride (98%) were purchased from Sigma-Aldrich. Tetrahydrofurane (>99.5%) was purchased from Bernd-Kraft. Hydrochloric acid (37 wt.-%) was purchased from Grüssig. The cross-coupling reaction was carried out under Ar atmosphere. Ar (purity 5.0) was passed through zeolite, P_2_O_5_, and the BTS catalyst column.

The equilibration of SMPAF-303 was carried out under an atmosphere with controlled relative humidity. The equilibration was carried out for 48 h at 33, 67, and 100% RH, using a saturated salt solution or water.

### 3.2. PAF-303

For the tetrakis-(4-bromophenyl)methane (Br-TPM), 477 mg (0.75 mmol, 1 eq.) was put into a flask with 251 mg (1.5 mmol, 2 eq.) 1,4-phenylenediboronic acid and 13.6 mg (32.5 µmol, 0.04 eq.) 1,1′-bis(diisopropylphosphino)ferrocene. A total of 37.6 mg (32.5 µmol, 0.04 eq.) tetrakis-(triphenylphosphino)palladium(0) was added, with 15 mL degassed 2 M K_2_CO_3_ solution and 130 mL THF under an inert gas atmosphere. The solution was boiled under reflux at 393 K for 24 h. When the reaction was complete the solid was filtered off, and washed with THF, EtOH, H_2_O, and 2 M HCl, three times. The beige solid was purified with EtOH Soxhlett and additionally dried under reduced pressure. Yield: 198 mg (0.42 mmol, 56%).

### 3.3. ClMPAF-303

Paraformaldehyde (503 mg, 16.7 mmol) was dissolved in 10 mL conc. HCl. Additionally, 1.5 mL conc. phosphoric acid, 3 mL acetic acid, 124 mg (0.9 mmol) ZnCl_2_, and 101 mg (215 µmol) PAF-303 were immediately added, and the high-pressure flask was sealed. The suspension was heated up to 383 K for five days, filtered off, purified by Soxhlett (1:1, EtOH:H_2_O), and dried under reduced pressure. Yield: 103 mg (200 µmol, 93%).

### 3.4. SMPAF-303

To synthesize SMPAF-303, 60 mg (105 µmol) of ClMPAF-303 was added to 20 mg (161 mmol) Na_2_SO_3_ and 12.15 mL water in a hydrothermal high-pressure vessel and heated up to 473 K, for five days. The resulting framework was protonated by 1 M H_2_SO_4_ and washed with water. Yield: 43.5 mg (77 µmol, 73%).

### 3.5. Methods

IR measurements were carried out on a JASCO FT/IR-6100 Fourier-transform IR spectrometer with an attenuated total reflectance unit. Ar physisorption measurements were carried out on an Autosorb I at 87 K with Ar as adsorbate. Water physisorption measurements were carried out at 293 K on an Autosorb IQ-MP-MP-AG setup (Anton Paar, QuantaTec, BoyntonBeach, FL, USA). All samples were degassed for 12 h at 393 K under reduced pressure. The measurements were analyzed with the software package ASIQ ver. 3.0. CHNS analysis was carried out on an ELEMENTAR UNICUBE with sulfanilamide as a reference. Powder X-ray diffraction measurements were carried out on a STOE STADI P diffractometer with a Ge(111)monochromator and Cu-K_α_ radiation. For the measurement, the frameworks were placed in 1.0 mm capillaries and measured at RT in Debye–Scherrer geometry. Thermogravimetric analysis (TGA) was measured on a Mettler Toledo TGA/SDTA851e under air atmosphere in the temperature range from 303 to 973 K with a heating rate of 10 K min^−1^. The differential scanning calorimetry (DSC) measurements were carried out on a Mettler Toledo DSC/SDTA 821e DSC with an autosampler in the temperature range from 193 to 353 K with 5 K min^−1^ under a nitrogen atmosphere with three heating cycles. For the evaluation, the third cycle was used. The samples were equally saturated at 33, 67, and 100%, as described above. The pH values for the titration curves were measured with a Mettler Toledo seven2go pH meter. The titration was carried out with an aqueous 0.8163 mM NaOH solution.

X-ray photoelectron spectroscopy (XPS) measurements were made with a PHI 5000 VersaProbe III system fitted with an AI Ka excitation source (hν = 1486.6 eV) and a dual neutralizer (electron and Ar+ gun) at 10-10 mbar. The source diameter of the X-ray beam was 100 µm. The multichannel analyzer collected the corresponding photoemission with 45° take-off angle. The pass energies of 292.5 and 69 eV were used for the measured spectra. The analysis of the spectra was carried out with a Multipak software pack version 9.9 provided by the manufacturers. The 13C single-pulse solid-state NMR spectra were measured on a Bruker Avance III HD spectrometer with a B0 field of 9.4 T. The pulse length was 3.20 µs, and the recycle delay was set to 120 s. The ^1^H-^1^H double-quantum (DQ) single-quantum (SQ) spectra were recorded on a Bruker Avance III spectrometer operating at a magnetic field strength of 14.1 T, using an R18_8_^5^ (ν(^1^H) ≈ 62.5 kHz) recoupling sequence with an S_0_S_π_ supercycle. The DQ excitation time was set to multiples of one RR’ block (22.22 µs): 111.11, 155.56, 2000.00, and 244.44 µs, and a recycle delay of 2.0 s was chosen.

Impedance measurements were made on a Novocontrol Alpha Analyzer with ZG4 and a 5 mV AC voltage was applied. The frequency range was set to 4 MHz to 1 Hz. The samples were placed between two steel electrodes to build a capacitor-like setup. SMPAF-303 was equilibrated under controlled relative-humidity gas flow for one day. The sample was conducted with the metal electrodes and the pressure was set to about 200 bar. The relative humidity of the atmosphere was constant during the measured temperature range. The equilibration time for every temperature step was 30 min. After the measurement, the length l and conduction area A were determined. The conductivity was calculated according to the formula σ = l(π·r^2^·R)^−1^, r = 4 mm. R is the resistance from the high-frequency semicircle of the Nyquist plot (Cole plot). The resistance was simulated with the equivalent circuit (Figure S7, ESI) in RelaxIS 3.

## 4. Conclusions

In this work, we report on the synthesis of an innovative new material for proton conduction. The synthesis route follows two post-synthetic modifications. The first step is the addition of a CH_2_-Cl side-chain unit via Blanc reaction to ClMPAF-303. The second one is the substitution of the chlorine by a sulfonic acid group by functional group interconversion to SMPAF-303. Introducing the CH_2_-SO_3_H side chains into the microporous framework increases its ability to adsorb water. For 100% RH, the moderate grafting density of one side chain per repeating unit led to λ of 10 H_2_O/SO_3_^−^. Water adsorption and proton conductivities measured as a function of the water uptake suggest that the framework shows an alternation in polarity. The strong interactions between water molecules and the sulfonic acid group increase the polarity, whereas the aromatic backbone limits the affinity for water. This restricts the free diffusion of oxonium ions through the pores, particularly for low RH. Only at high relative humidities are the pores completely filled, which ensures free diffusion of the oxonium ions and high conductivity, as for Nafion^®^. Due to the irreversibly bonded SO_3_H groups, SMPAF-303 is a promising framework for proton-conducting materials, which warrants further studies to increase its IEC and conductivity.

## Figures and Tables

**Table 1:** Stoichiometry of PAF derivatives calculated from Tables S1–S4 (ESI).

Framework	Stoichiometry
PAF-303	C_37_H_24_Br_0.6_
ClMPAF-303	C_38_H_25_Br_0.6_Cl_1.2_ × H_2_O
SMPAF-303	C_38_H_25_Br_0.2_SO_3_H × 3 H_2_O

**Table 2:** Ar surface area, total pore volume, and micropore ratio of PAF-303 and its derivatives. * Calculated from the adsorption branch using the BET equation. ** Derived using a QSDFT kernel of the adsorption branch.

Sample	BET SA(Ar)/m^2^ g^−1^ *	DFT SA(Ar)/m^2^ g^−1^ **	Pore Volume/cc g^−1^ **	Micropore Ratio/% **
PAF-303	1317	1302	0.681	74
ClMPAF-303	1132	1155	0.539	78
SMPAF-303	743	773	0.337	82

* Calculated using the BET equation. ** Derived using a QSDFT kernel.

## Data Availability

Data can be supplied by petition.

## Supplementary Materials

The following supporting information can be downloaded at: https://www.mdpi.com/article/10.3390/molecules29071666/s1, Figure S1: IR spectra of BDB, BrTPM, PAF-303, ClMPAF-303, and SMPAF-303; Table S1: Ratio of the NMR integrals from deconvolution (Figure 1a); Table S2: Composition of PAF-303; Figure S2: Powder XRD diffractogram of PAF-303, ClMPAF-303, and SMPAF-303; Table S3: Composition of ClMPAF-303; Table S4: Composition of SMPAF-303; Figure S3: XPS spectrum of ClMPAF-303; Figure S4: Titration curves of SMPAF-303; Table S5: Experimental data of the titration curves; Figure S5: TGA measurements of PAF-303, ClMPAF-303, and SMPAF-303; Table S6: Experimental data of DSC curves; Figure S6: Pore-size distribution and cumulative pore volume of PAF-303, ClMPAF-303, and SMPAF-303; Figure S7: Equivalent circuit for impedance measurements; Figure S8: Nyquist plot of impedance measurement of SMPAF-303 at 33% RH; Table S7: Fitting data of impedance measurement of SMPAF-303 at 33% RH; Figure S9: Nyquist plot of impedance measurement of SMPAF-303 at 67% RH; Table S8: Fitting data of impedance measurement of SMPAF-303 at 67% RH; Figure S10: Nyquist plot of impedance measurement of SMPAF-303 at 100% RH; Table S9: Fitting data of impedance measurement of SMPAF-303 at 100% RH.
