# Supplementary material for: Thermodynamically Stable Functionalization of Microporous Aromatic Frameworks with Sulfonic Acid Groups by Inserting Methylene Spacers
Source: Molecules. 2024 Apr 8;29(7):1666. doi: 10.3390/molecules29071666 (PMC11013227; doi:10.3390/molecules29071666)
Supplement: Supplementary file 1 [file molecules-29-01666-s001.zip › molecules-2937456-supplementary.pdf]

Supporting Information

Thermodynamically Stable Functionalization of Microporous Aromatic Frameworks with Sulfonic Acid Groups by Inserting Methylene Spacers

Simon F. Winterstein [1], Michael Bettermann [1], Jana Timm [2], Roland Marschall [2] and Jürgen Senker [1,*]

[1] Inorganic Chemistry III and Northern Bavarian NMR Centre, University of Bayreuth, Universitaetsstrasse 30, 95447 Bayreuth, Germany
[2] Physical Chemistry III, Department of Chemistry, University of Bayreuth, Universitaetsstr. 30, 95447 Bayreuth, Germany; roland.marschall@uni-bayreuth.de (R.M.)
* Correspondence: juergen.senker@uni-bayreuth.de

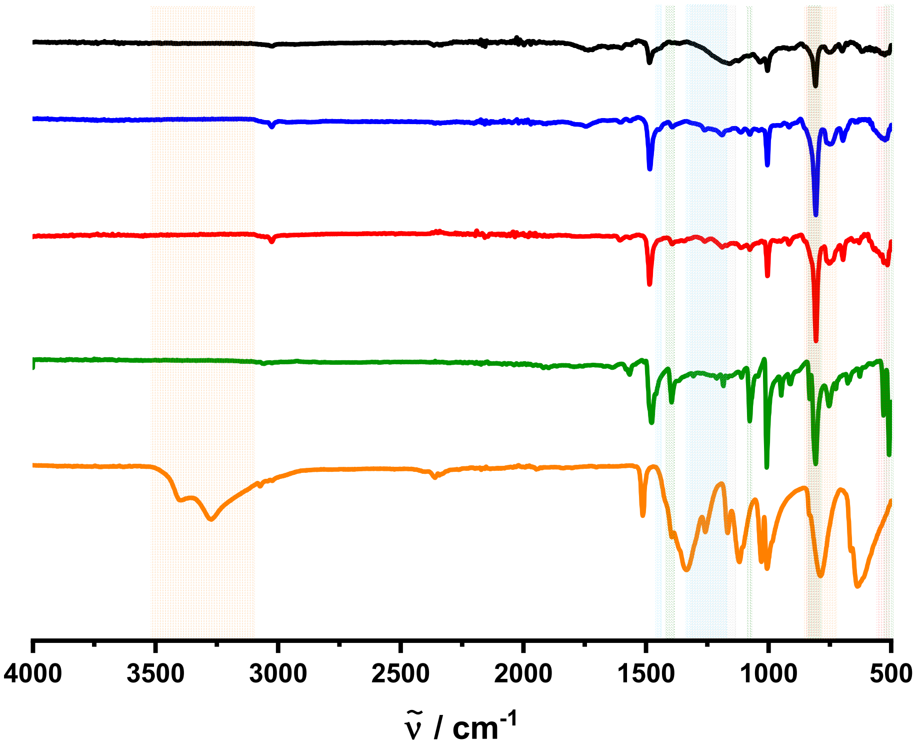

Figure S1: IR spectra of BDB (orange), BrTPM (green), PAF-303 (red), ClMPAF-303 (blue), and SMPAF-303 (black) with characteristic bands in the corresponding color.

Table S1: Ratio of the NMR integrals from deconvolution (Figure 1a). The ratio is normalized to the carbon atoms of the structure. The stoichiometry calculated by NMR is equal to the integrals because the C-1 integral represents the repeating unit with 37 or 38 C atoms.

framework		*C*	*Br*	*Cl*	*S*
PAF-303	Integral C-1 to C-Br	1	0.60		
	Normalized to 37 C atoms at.-%	37	0.6		
ClMPAF-303	Integral C-1 to C-Br, CH_2-Cl[a] and **C**-CH_2[b]	1	0.67	1.09[a]/1.79[b]	
	Normalized to 38 C atoms at.-%	38	0.7	1.1[a]/1.8[b]	
SMPAF-303	Integral C-1 to C-Br, CH2-S[a] and **C**-CH_2[b]	1	0.34		1[c]/0.91[b]
	Normalized to 38 C atoms at.-%	38	0.3		1[c]/0.9

[a] *Integral of C-Cl, which is overlapping with the quaternary carbon C-1*
[b] *Integral of the aromatic carbon at which the side chain is connected to*
[c] *Integral of C-S, which is overlapping with the quaternary carbon C-1*

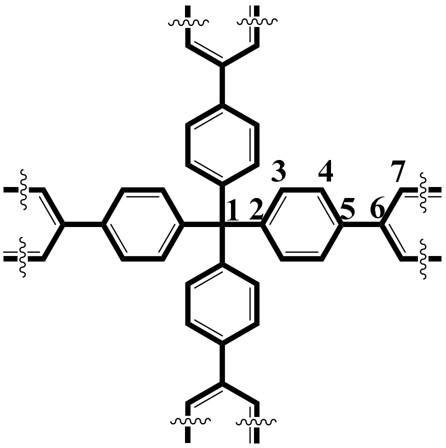

Table S2: Composition which was calculated from EA, EDX, and NMR of PAF-303. The composition was normalized to 37 C atoms that correspond to the repeating unit of PAF-303. The bromine value normalized to 37 C determined from NMR is the same as the integral from C-Br normalized to the integral of C-1. C-1 represents one repeating unit with 37 C.

PAF-303		*C*	*H*	*N*	*S*	*Br*
Composition	**at.-%**	**37**	**24**	**--**	**--**	**0.6**
EA	wt.-%	82.95	4.56	0.12	0.15	--
	at.-%	6.91	4.53	0.01	0.00	--
	Normalized to 37 C atoms at.-%	37	24.26	0.05	0.03	--
EDX	at.-%	91.78				1.26
	Normalized to 37 C atoms at.-%	37				0.5

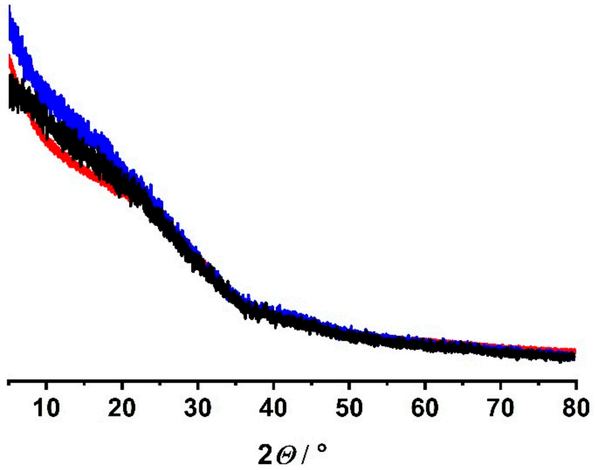

Figure S2: Powder X-ray diffractogram of PAF-303 (red), ClMPAF-303 (blue), and SMPAF-303 (black).

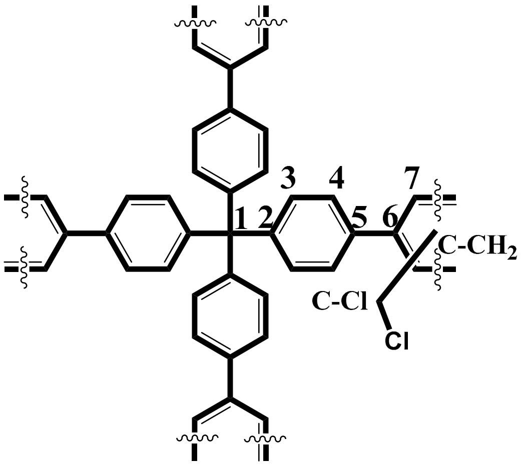

Table S3: Composition which was calculated from EA, EDX, and NMR for ClMPAF-303. The composition was normalized to 38 C atoms that correspond to the repeating unit of ClMPAF-303.

ClMPAF-303		*C*	*H*	*N*	*S*	*Br*	*Cl*
Composition	**at.-%**	**38**	**26.9**	**--**	**--**	**0.6**	**1.2**
EA	wt.-%	80.38	4.78	0.12	0.37	--	
	at.-%	6.69	4.74	0.02	0.15	--	
	Normalized to 38 C atoms at.-%	38	26.9	0.1	0.1	--	
EDX	at.-%	90.07				1.25	1.43
	Normalized to 38 C atoms at.-%	38				0.5	0.6

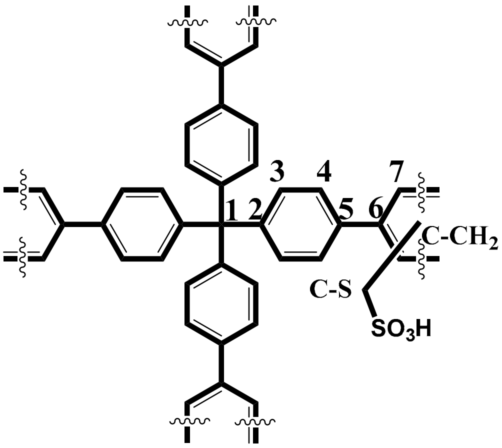

Table S4: Composition which was calculated from EA, EDX, and titration. The composition was normalized to 38 C atoms that correspond to the repeating unit of SMPAF-303.

SMPAF-303		*C*	*H*	*N*	*S*	*Br*	*Cl*
Composition	**at.-%**	**38**	**31.9**	**--**	**1**	**0.2**	**--**
EA	wt.-%	67.92	4.78	0.35	4.96	--	--
	at.-%	5.65	4.74	0.02	0.15	--	--
	Normalized to 38 C atoms at.-%	38	31.9	0.2	1.0	--	--
EDX	at.-%	79.3	--	--	2.3	0.3	--
	Normalized to 38 C atoms at.-%	38	--	--	1.1	0.1	--
Titration	Normalized to 38 atoms at.-%	38	--	--	1.0	--	

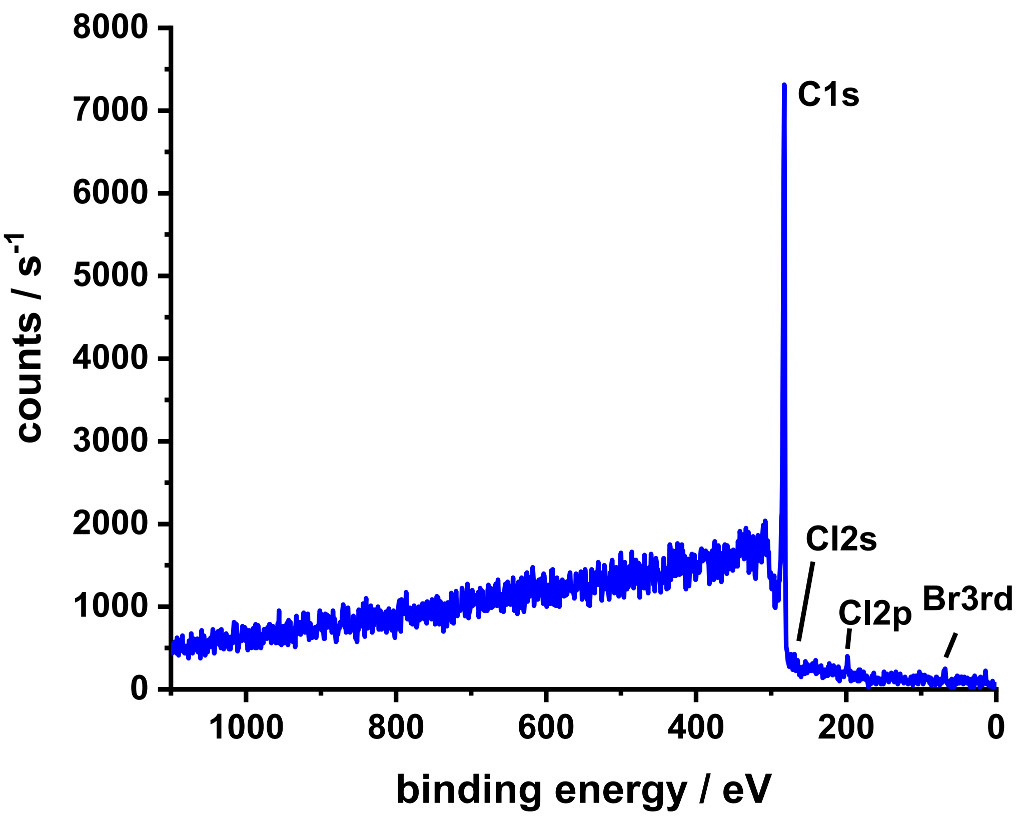

Figure S3: XP spectrum of ClMPAF-303 after sputtering the sample with Ar.

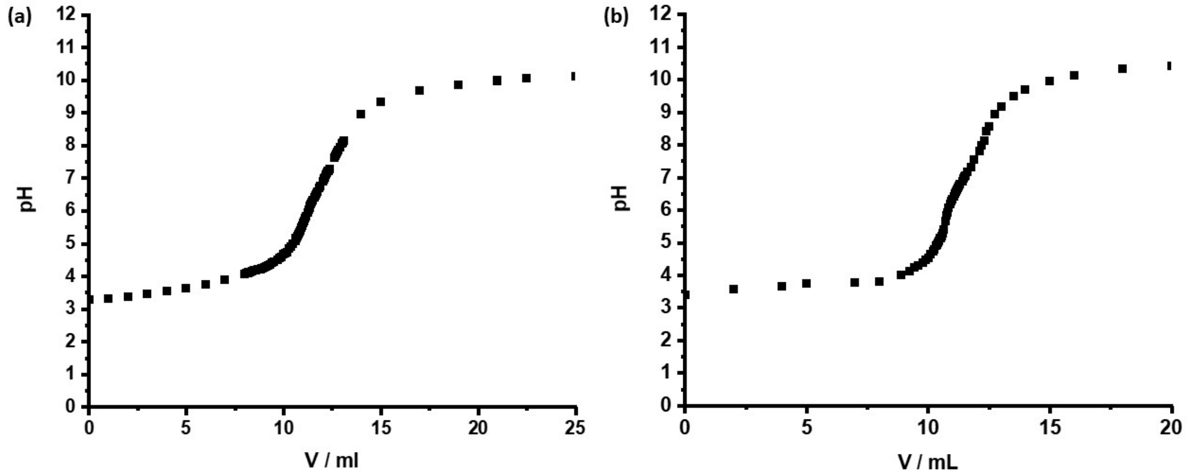

Figure S4: Titration curve of SMPAF-303 with 0.8163 mM NaOH solution after synthesis (a) and after characterization and stability test of the framework (b) To test the irreversible bond formation of C-S, the framework was stirred in 1 M HCl at 253 K for 24 h. The framework was filtered off, washed with water and the SO_3H groups were determined.

Table S5: Experimental data according to Figure S5, sulfonation degree x and ion exchange capacity (ICE) calculated from titration.

sample	weight / mg	V_{EP} / ml	X / %	ICE
SMPAF-303 after synthesis	5.5	11.63	100	1.73
SMPAF-303 after experiments	5.4	11.47	100	1.73

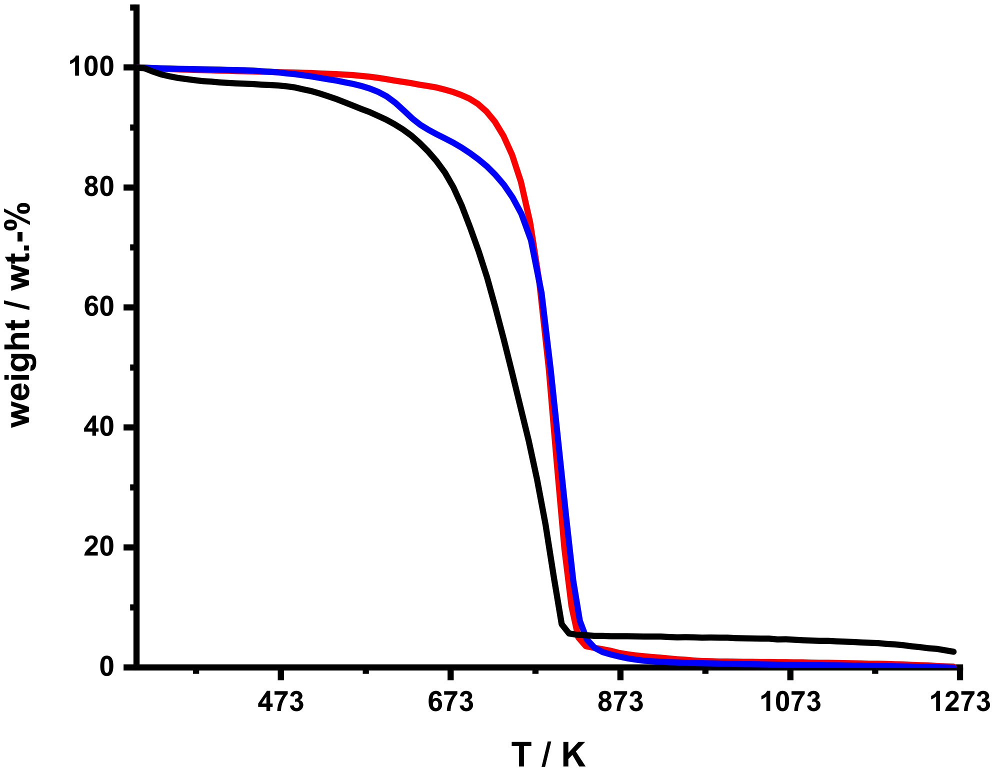

Figure S5: TGA measurements of PAF-303 (red), ClMPAF-303 (blue), and SMPAF-303 (black).

Table S6: Onset of melting point and melting enthalpy of SMAPF-2 from DSC curve (figure 2b).

framework	33 %-rh		67 % rh		100 %	
	T_m / K	$\Delta_m H$ / J	T_m / K	$\Delta_m H$ / J	T_m / K	$\Delta_m H$ / mJ
SMPAF-303	--	--	--	--	256	26.9

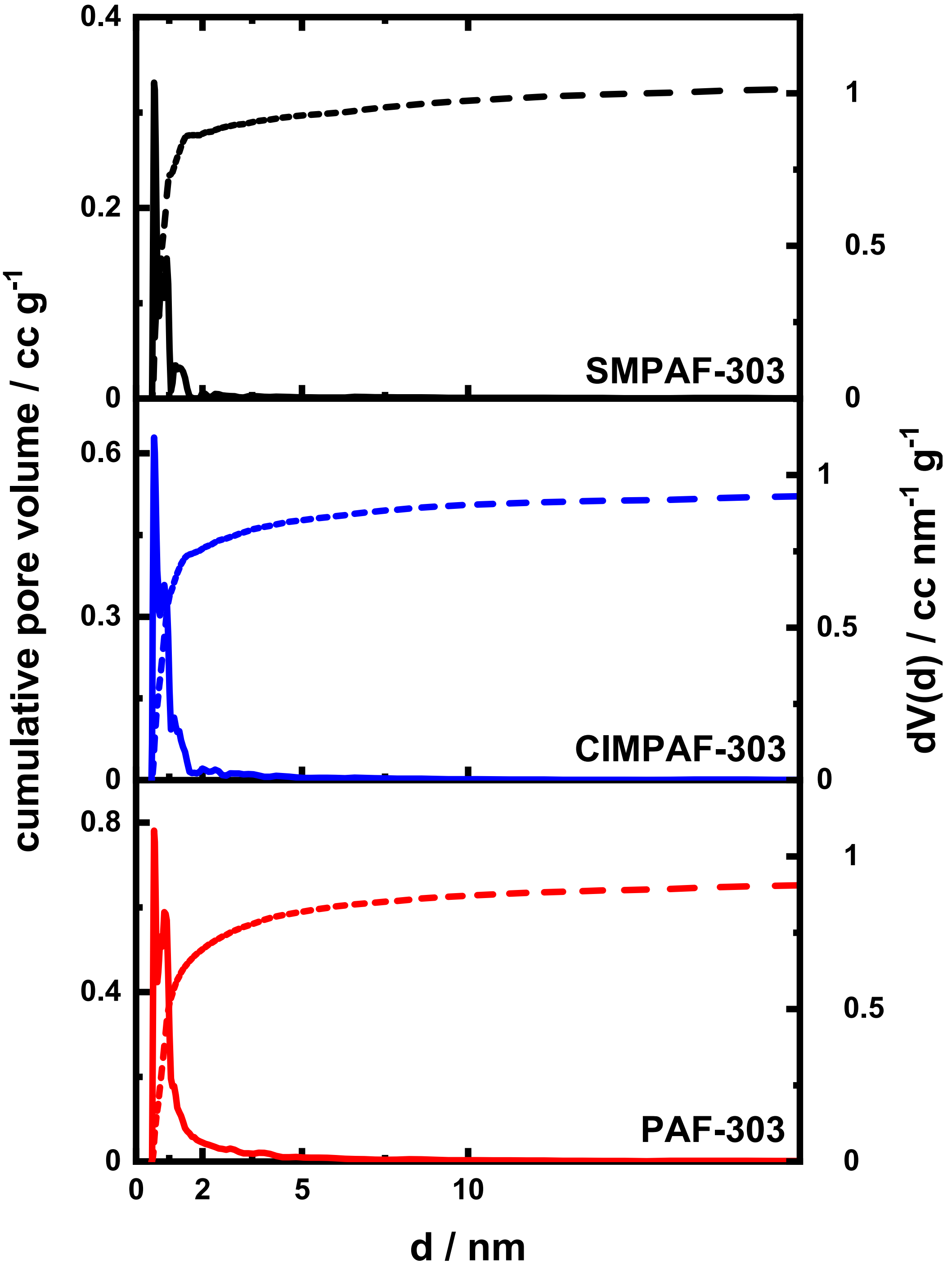

Figure S6: Pore size distribution (lined) and cumulative pore volume (segmented) calculated with DFT simulation (kernal: Ar at 87 K on carbon QSDFT (slit pore, QSDFT equilibrium model)) where the adsorption branches were used from the Ar isotherms of Figure 1b.

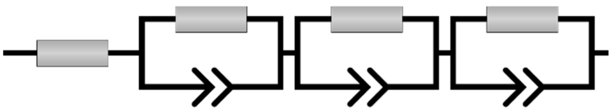

Figure S7: General equivalent circuit for impedance measurements of SMPAF-303, containing a preliminary resistance (R_E) for the electrical conductivity and three in-series connected resistances (R) - constant-phase-elements (CPE).

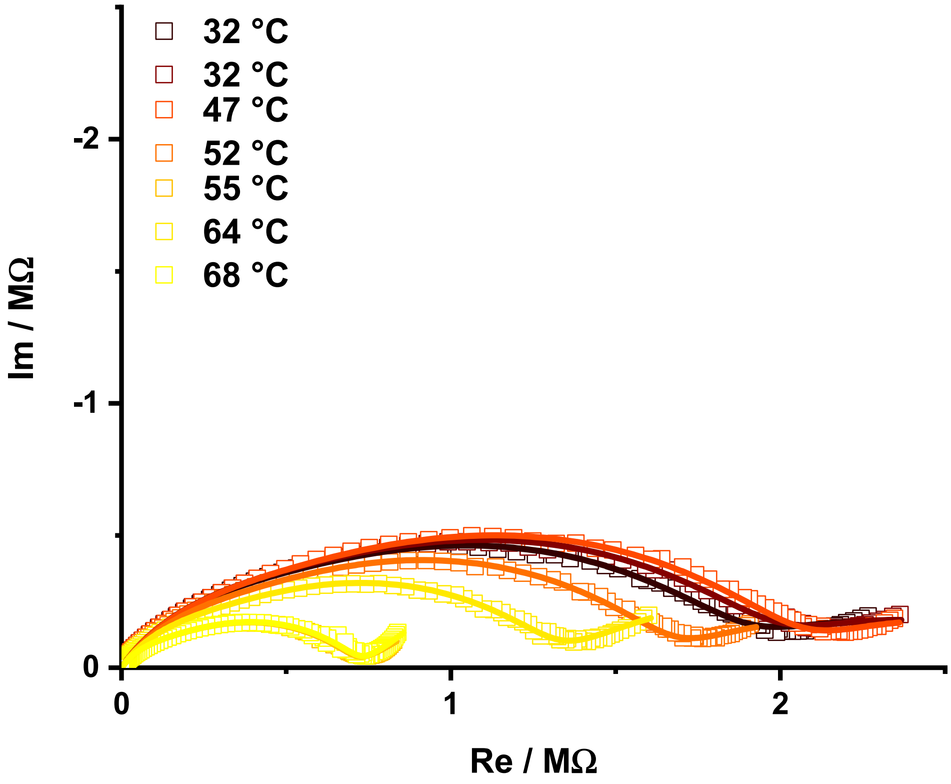

Figure S8: Nyquist plot of SMPAF-303 at 33 % RH with an enlarged image section of the proton conductivity.

Table S7: Simulation parameters of SMPAF-303@33 % RH.

T / *K*	bulk			grain boundaries			electrode interface		
	R_B / kΩ	CPE_B / 10^{-9} S	α_B	R_{GB} / MΩ	CPE_{GB} / 10^{-9} S	α_{GB}	R_{EI} / kΩ	CPE_{EI} / 10^{-6} S	α_{EI}
305	363 (90)	1.56 (27)	0.74 (3)	1.50 (15)	6.60 (43)	0.64(3)	856(278)	1.55(20)	0.47(11)
305	390 (96)	1.58 (27)	0.74 (3)	1.57 (16)	6.58 (43)	0.64(3)	877(302)	1.55(20)	0.47(12)
320	350 (83)	1.57 (30)	0.74 (3)	1.69 (14)	6.51 (39)	0.63(2)	891(516)	2.26(28)	0.46(16)
325	265 (62)	1.61 (33)	0.75 (3)	1.39 (11)	6.92 (41)	0.63(2)	943(658)	2.87(27)	0.45(15)
328	104 (18)	1.83 (42)	0.76 (3)	0.60(2)	9.98 (31)	0.62(1)	1E12(--)	7.46(30)	0.41(2)
337	219 (46)	1.73 (33)	0.74 (3)	1.09(7)	7.77 (41)	0.63(2)	138(878)	2.79(16)	0.45(10)
341	116 (32)	2.10 (69)	0.74 (4)	0.61(4)	10.4 (5)	0.62(2)	1E12(--)	6.68(33)	0.48(3)

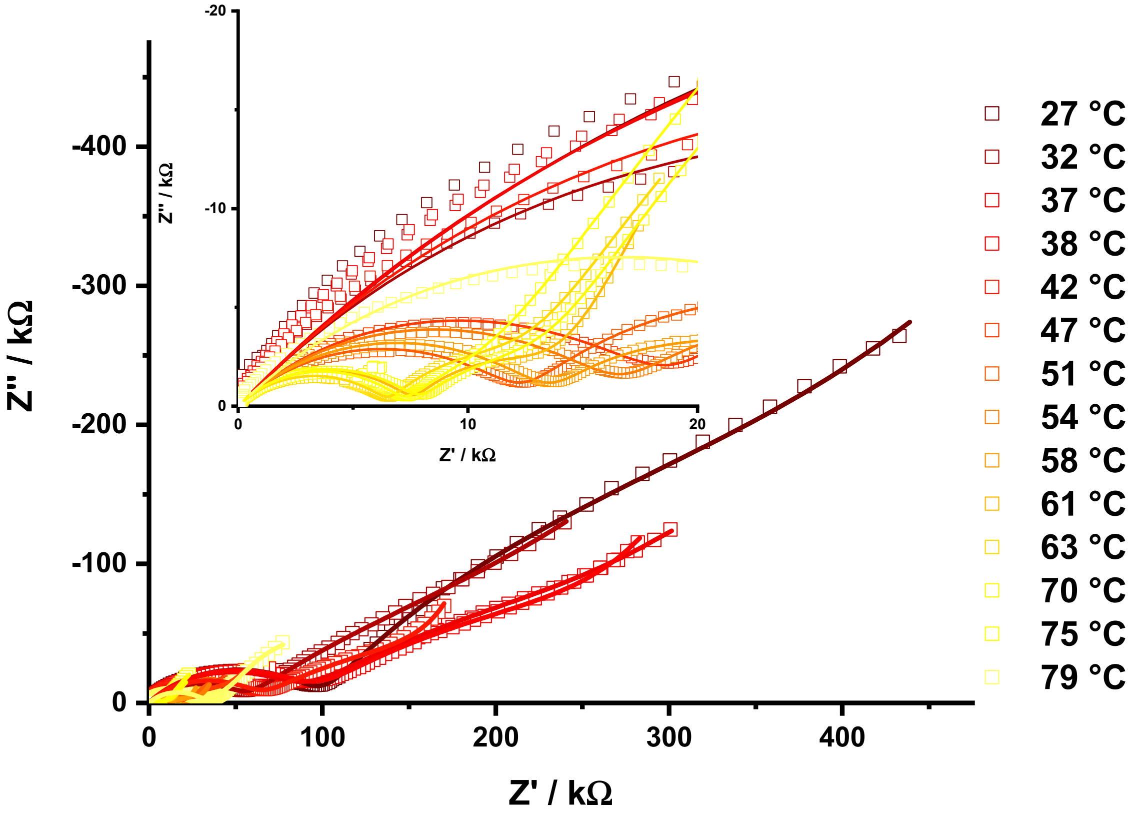

Figure S9: Nyquist plot of SMPAF-303 at 67 % RH with an enlarged image section of the proton conductivity.

Table S8: Simulation parameters of SMPAF-303@67 % RH.

T / *K*	bulk			grain boundaries			electrode interface		
	Z_B / kΩ	CPE_B / 10^{-8} S	α_B	R_{GB} / kΩ	CPE_{GB} / 10^{-5} S	α_{GB}	R_{EI} / 10^{11} Ω	CPE_{EI} / 10^{-5} S	α_{EI}
299	133(2)	1.63(8)	0.56(0)	58.7(8.0)	0.44(2)	0.76(3)	5.51	1.00(8)	0.34(2)
300	101(1)	1.86(14)	0.55(1)	357(53)	0.19(1)	0.62(1)	1E4	0.67(10)	0.72(6)
305	54.7(1.1)	2.76(35)	0.57(1)	235(100)	0.41(5)	0.48(2)	1E4	1.37(59)	0.66(13)
310	85.9(1.4)	2.32(17)	0.57(1)	367(85)	0.27(2)	0.46(2)	3.01E-6	2.77(1.23)	1.00(19)
311	94.5(1.1)	2.58(17)	0.56(1)	253(22)	0.25(1)	0.50(1)	9.58E3	2.05(23)	0.89(5)
315	63.6(1.1)	2.69(27)	0.57(1)	162(22)	0.44(3)	0.47(2)	1E4	3.73(5.52)	1.00(8)
320	18.8(1)	6.63(38)	0.54(0)	34.9(2.7)	0.92(3)	0.56(1)	1.70E-6	7.02(59)	0.83(7)
324	12.4(1)	6.90(36)	0.55(0)	19.2(6)	1.25(4)	0.58(1)	3.08E-7	11.9(5)	0.99(3)
327	16.8(1)	6.61(23)	0.54(0)	14.1(3)	1.07(3)	0.59(1)	1E4	10.9(2)	0.75(1)
331	13.7(1)	6.38(25)	0.55(0)	9.99(25)	1.26(4)	0.60(1)	1E4	12.9(2)	0.77(1)
334	7.70(5)	7.52(60)	0.56(1)	6.33(24)	1.66(10)	0.65(2)	1E4	14.9(3)	0.75(1)
336	6.58(5)	8.98(83)	0.55(1)	6.55(59)	2.18(15)	0.63(2)	1.21E-6	11.1(3)	0.76(4)
343	7.94(6)	7.88(64)	0.55(1)	6.20(58)	2.30(20)	0.59(2)	1.18E-6	8.35(19)	0.77(3)
348	7.28(7)	6.78(70)	0.57(1)	4.01(82)	3.56(56)	0.57(4)	2.62E-6	5.73(12)	0.71(3)

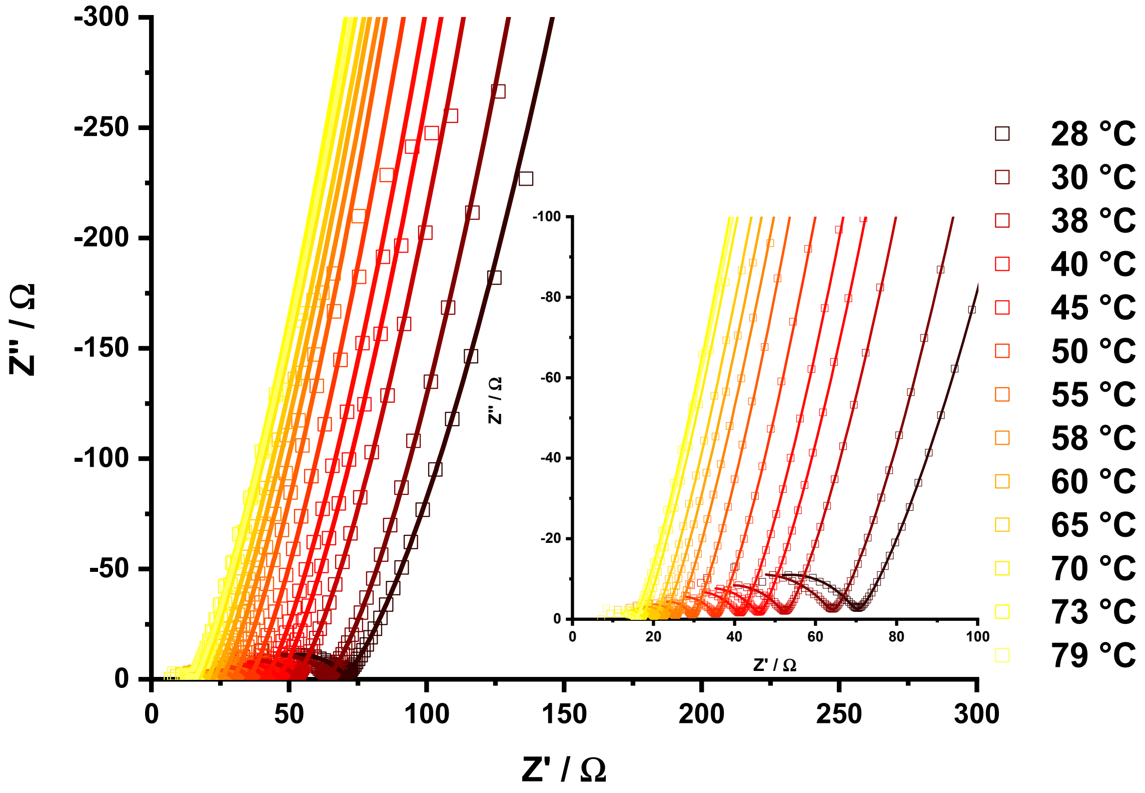

Figure S10: Nyquist plot of SMPAF-303 at 100 % RH with an enlarged image section of the proton conductivity.

Table S9: Simulation parameters of SMPAF-303@100 % RH.

T / *K*	**elec-trode**	**bulk**			**grain boundaries**			**electrode interface**		
	R_E / Ω	R_B / Ω	CPE_B / 10^{-8} S	α_B	R_{GB} / Ω *	CPE_{GB} / 10^{-3} S	α_{GB}	R_{EI} / Ω *	CPE_{EI} / 10^{-5} S	α_{Ei}
301	38.7(7)	30.4(9)	3.04(56)	0.79(1)	80.6	1.29(28)	0.44(2)	1E15	1.56(30)	0.89(5)
303	30.1(7)	33.4(8)	5.41(65)	0.73(1)	54.0	0.94(12)	0.49(1)	7E14	1.16(9)	0.91(2)
311	27.9(6)	23.9(7)	4.66(66)	0.77(1)	77.3	1.23(58)	0.47(3)	1E15	1.17(21)	0.91(5)
313	23.7(5)	21.7(6)	4.62(64)	0.77(1)	46.1	1.48(22)	0.47(1)	2E13	1.24(10)	0.91(2)
318	21.6(5)	19.3(5)	5.07(67)	0.77(1)	42.0	1.52(22)	0.47(1)	1E13	1.27(9)	0.91(2)
323	20.1(6)	14.6(7)	3.29(80)	0.81(2)	70.6	2.35(332)	0.44(10)	4E12	1.34(54)	0.91(13)
328	18.6(5)	10.3(6)	2.06(70)	0.87(3)	90.1	3.91(458)	0.41(9)	1E13	1.53(121)	0.90(37)
331	16.3(4)	8.49(51)	1.92(72)	0.88(3)	963	5.49(282)	0.39(5)	3E12	1.84(7)	0.89(1)
333	15.2(3)	6.50(45)	9.15(44)	0.95(3)	4E6	0.02(1)	0.88(1)	3E12	988(608)	0.34(6)

338	14.0(3)	5.57(43)	1.12(61)	0.95(4)	2E3	9.76(686)	0.35(7)	8E14	2.08(9)	0.88(1)
343	12.0(3)	4.91(52)	2.48 (151)	0.91(4)	6.81	3.10(293)	0.48(9)	1E15	2.22(15)	0.87(1)
346	11.8(3)	3.49(41)	1.16 (127)	1.00(7)	5E3	0.17(6)	0.93(9)	1E15	102(250)	0.55(18)
352	12.2(2)	3.46(38)	1.12 (108)	1.00(6)	5E3	0.17(99)	0.93(36)	266	109(247)	0.54(18)

*No fitting error is reported because of the fitting error larger than 100 %.
